# Early stage of evolution of *Gonomyia* (Diptera: Limoniidae), new significant discoveries in the Cretaceous Iberian and Kachin amber

**DOI:** 10.1038/s41598-022-25803-0

**Published:** 2022-12-07

**Authors:** Iwona Kania-Kłosok, Antonio Arillo, Michał Tuchowski, Qingqing Zhang, Wiesław Krzemiński

**Affiliations:** 1 Department of Biology, Institute of Biology and Biotechnology, University of Rzeszów, Rzeszów, Poland; 2 Departamento de Biodiversidad, Ecología y Evolución, Facultad de Biología, Universidad Complutense, 28040 Madrid, Spain; 3 State Key Laboratory of Palaeobiology and Stratigraphy, Nanjing Institute of Geology and Palaeontology, Chinese Academy of Sciences, No. 39, East Beijing Road, Nanjing, 210008 People’s Republic of China; 4 Institute of Geosciences, University of Bonn, 53115 Bonn, Germany; 5 Institute of Systematics and Evolution of Animals, Polish Academy of Sciences, Kraków, Poland

**Keywords:** Evolution, Zoology

## Abstract

Thanks to detailed studies of inclusions in Spanish and Kachin amber, it was also possible to study the morphology of insects belonging to the genus *Gonomyia*. A new material under investigation made it possible to establish two new nominative for science subgenera within the genus *Gonomyia* has been designated with unique set of characters of antenna, wing venation and genitalia. Two new species within two new subgenera have been described and documented by drawings and photographs, there are *Gonomyia* (*Iberiana*) *penalveri* subgen. et sp. nov. and *Gonomyia* (*Cretagonomyia*) *burmitica* subgen. et sp. nov. The new discovery is the first record of the genus *Gonomyia* (Diptera: Limoniidae) in Cretaceous Spanish amber and the second in Kachin amber. The new discovery adds to the knowledge of the crane fies’ diversity and evolution, especially its first stage in the Cretaceous.

## Introduction

The Cretaceous was the time represented either archaic or recent forms and in many respects an intermediate period between the Mesozoic and the Cenozoic eras. To understand the functioning of modern ecosystems, we must go back to the Cretaceous period and learn about the relationship between organisms living at that time. Such possibilities are offered by studies on Cretaceous Spanish and Kachin amber. The large variety and number of insect inclusions preserved in Kachin amber, especially the flies, is unique and allows us to recreate some of the details of ancient ecosystems.

The genus *Gonomyia* Meigen^1^ (Limoniidae, Chioneinae) is represented in recent fauna by over 600 species and subspecies belonging to nine subgenera^2^ as *Gonomyia*, *Gonomyina* Alexander^3^; *Idiocerodes* Savchenko^4^, *Leiponeura* Skuse^5^, *Megalipophleps* Alexander^6^, *Neolipophleps* Alexander^7^, *Paralipophleps* Alexander^7^, *Prolipophleps* Savchenko^4^ and *Teuchogonomyia*, Alexander^8^. Within them the most diversified are subgenera *Leiponeura* and *Gonomyia*, with 327 and 190 species and subspecies^2^. In fossil record four subgenera are represented as *Gonomyia*, *Paralipophleps*, *Electrogonomyia* Alexander^9^ and *Azaria* Kania, Krzemiński and Krzemińska^10^, the last two being known only from the fossil record. Nine species of *Gonomyia* known from fossil record remain unplaced in any subgenus. The oldest species of *Gonomyia*—*Gonomyia* (*Azaria*) *libanensis* Kania, Krzemiński and Krzemińska^10^ is known from Cretaceous Lebanese amber dated on Barremian^11^, the oldest Chioneinae are known since Lower Cretaceous^10^. Additionaly, one species of *Gonomyia* was mentioned from Upper Cretaceous Kachin amber^12^. Two other species of this genus were described from Eocene Baltic amber and classified into two different subgenera—*Electrogonomyia* and *Gonomyia*^9^. Four species of *Gonomyia* are known from Eocene/Oligocene of England, two from Oligocene of Germany, and two from Eocene and Miocene of Italy (imprints in sediments), two others from Dominican amber—*Gonomyia* (*Paralipophleps*) *asymmetrica*^13^ and one representative of *Gonomyia* unplaced to any species^14^. In total, 14 species of *Gonomyia* have been described from fossil record (Table 1).

**Table 1:** List of fossils belonging to genus *Gonomyia*, age and localities.

Species	Epoch	Type of material	Locality
*Gonomyia* (*Paralipophleps*) *asymmetrica* Podenas and Poinar^13^	Miocene	Inclusion/Dominican amber	Dominican Republic
*Gonomyia andrea* Krzemiński and Gentlini^15^	Miocene	Imprint	Italy
*Gonomyia* sp. Krzemiński^14^	Oligocene/Miocene	Inclusion/Dominican amber	Dominican Republic
*Gonomyia sturi* Heyden^16^	Oligocene	Imprint	Germany
*Gonomyia munda* Statz^17^	Oligocene	Imprint	Germany
*Gonomyia lutescens* Cockerell^18^	Eocene/Oligocene	Imprint	England
*Gonomyia indecisa* Cockerell and Haines^19^	Eocene/Oligocene	Imprint	England
*Gonomyia grisea* Cockerell^18^	Eocene/Oligocene	Imprint	England
*Gonomyia ferrea* Cockerell^18^	Eocene/Oligocene	Imprint	England
*Gonomyia* sp. Krzemiński and Krzemińska^20^	Eocene	Imprint	Italy
*Gonomyia* (*Gonomyia*) *oligocenica* Alexander^12^	Eocene	Inclusion/Baltic amber	Kaliningrad district
*Gonomyia* (*Electrogonomyia*) *pinetorum* Alexander^12^	Eocene	Inclusion/Baltic amber	Kaliningrad district
*Gonomyia* (*Gonomyia*) sp. Podenas and Poinar^11^	Upper Cretaceous	Inclusion/Kachin amber	Myanmar
*Gonomyia* (*Azaria*) *libanensis* Kania, Krzemiński and Krzemińska^9^	Lower Cretaceous	Inclusion/Lebanese amber	Lebanon

**Figure 1:**
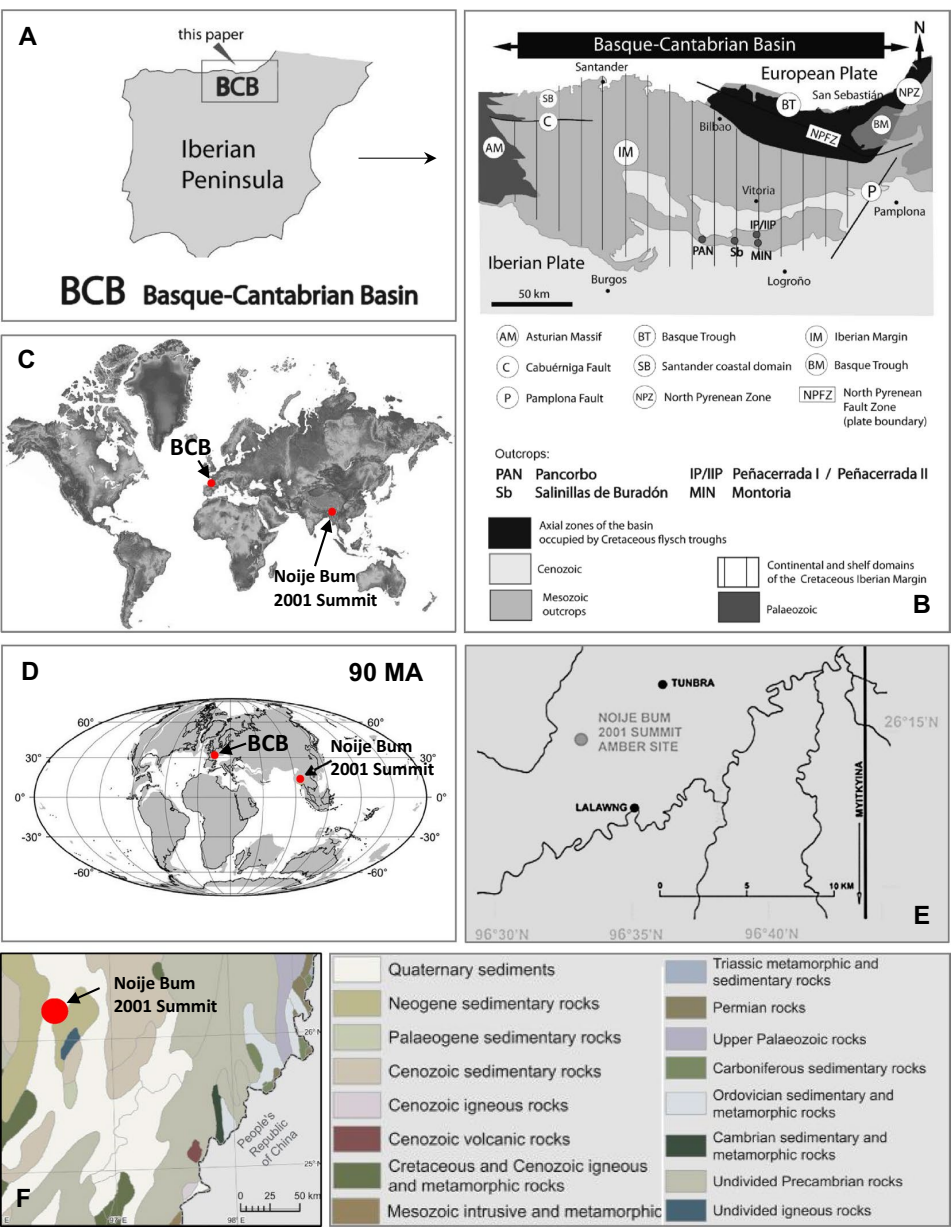
Maps with the position of Spanish and Kachin amber deposits localities. (**A**) Geographical setting of the studied section of Spanish amber deposits; (**B**) Geological setting with location of the studied section of Spanish amber deposits (after Barrón et al.^21^, modified); (**C**) Spanish and Kachin amber deposits localities; (**D**) Spanish and Kachin amber deposits localities in the Cretaceous; (**E**) Location of recent amber mining area in the Hukawng Valley, Myitkina Province, Myanmar; (**A**) Geological setting with location of the studied section of Kachin amber deposits. (**E**, **F**) Compiled from data provided by Kania et al.^22^.

Analysis of new fossil materials allowed to provide new information about Cretaceous (the oldest) stage of evolution of *Gonomyia*. There is no evidence that this group of insects existed on Earth earlier. It is the first evidence of the presence of this genus’ representatives—recorded as an inclusions in Cretaceous Spanish amber and the second from Kachin amber from Hukawng Valley, Myitkyina (Fig. 1C–F).

## Results

### Systematic palaeontology

**Order: Diptera** Linnaeus^23^.

**Infraorder: Tipulomorpha** Rohdendorf^24^.

**Family: Limoniidae** Rondani^25^.

**Subfamily: Chioneinae** Rondani^25^.

**Genus: *Gonomyia*** Meigen^1^.

Type species: *Limnobia tenella* Meigen^1^, by monotypy. [Originally proposed in synonimy; available by established use prior to 1961.]

**Subgenus: *Iberiana*** subgen. nov.

Type species: *Gonomyia* (*Iberiana*) *penalveri* subgen. et sp. nov.

*Diagnosis*. Antenna 10-segmented, shorter than palpus; last palpomere very elongate, as long as the previous three; Sc with its tip beyond half length of Rs; very elongate and almost straight Rs; short R_3_, approximately 0.3 × the length of R_4_, basal section of R_5_ separate Rs at almost right angle; the position of crossvein m-cu behind the bifurcation of Mb into M_1+2_ and M_3+4_ wherein closed; very small, only 1.5 × as long as wide, almost rhomboidal d-cell; A_2_ short, straight.

*Etymology*. The specific name is derived from Iberia. Gender: feminine.

*Description*. By monotypy, the description of the subgenus is the same as for the species.

*Remark*. Due to the morphology of the wing venation and morphology of antenna, it was possible to designate a new monotypic subgenus based on the female characteristics. Diversification of the number of segments of antennae due to sexual dimorphism is not observed in modern species belonging to the genus, and such variability has not been indicated in the fossil material. The monotypic subgenus *Azaria* known from the Cretaceous period characterize by d-cell open by atrophy of the basal section of vein M_3_ and by the position of cross-vein m-cu far beyond the bifurcation of Mb into M_1+2_ and M_3+4_. In *Iberiana* subgen. nov. cross-vein m-cu occur far behind the fork of Mb like in subgenus *Azaria*, but d-cell in *Iberiana* subgen. nov. is closed, vein A_2_ is short and straight, while in subgenus *Azaria* this vein is elongate and waved. The number of antennal segments in *Iberiana* subgen. nov. is unique.

### *Gonomyia* (*Iberiana*) *penalveri* subgen. et sp. nov

(Figs. 2, 3, 4, 5).

**Figure 2:**
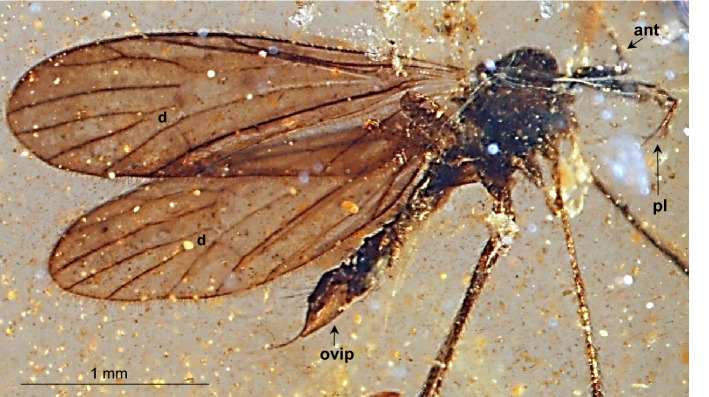
*Gonomyia* (*Iberiana*) *penalveri* subgen. et sp. nov. (Limoniidae), inclusions in Spanish amber; body, lateral view.

**Figure 3:**
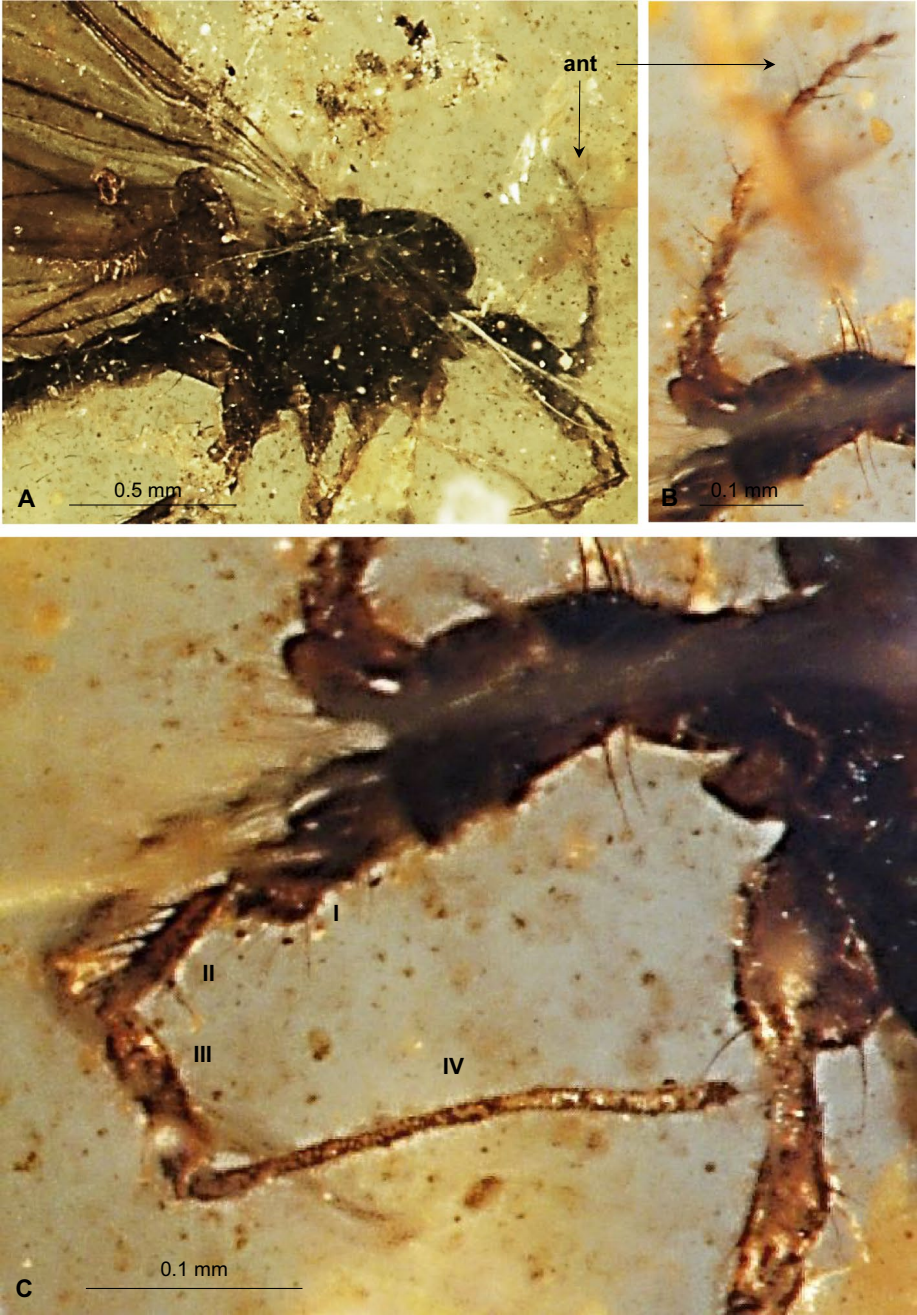
*Gonomyia* (*Iberiana*) *penalveri* subgen, et sp. nov. (Limoniidae), inclusions in Spanish amber: (**A**) head and thorax, lateral view; (**B**) antennae, lateral view; (**C**) palpi lateral view.

**Figure 4:**
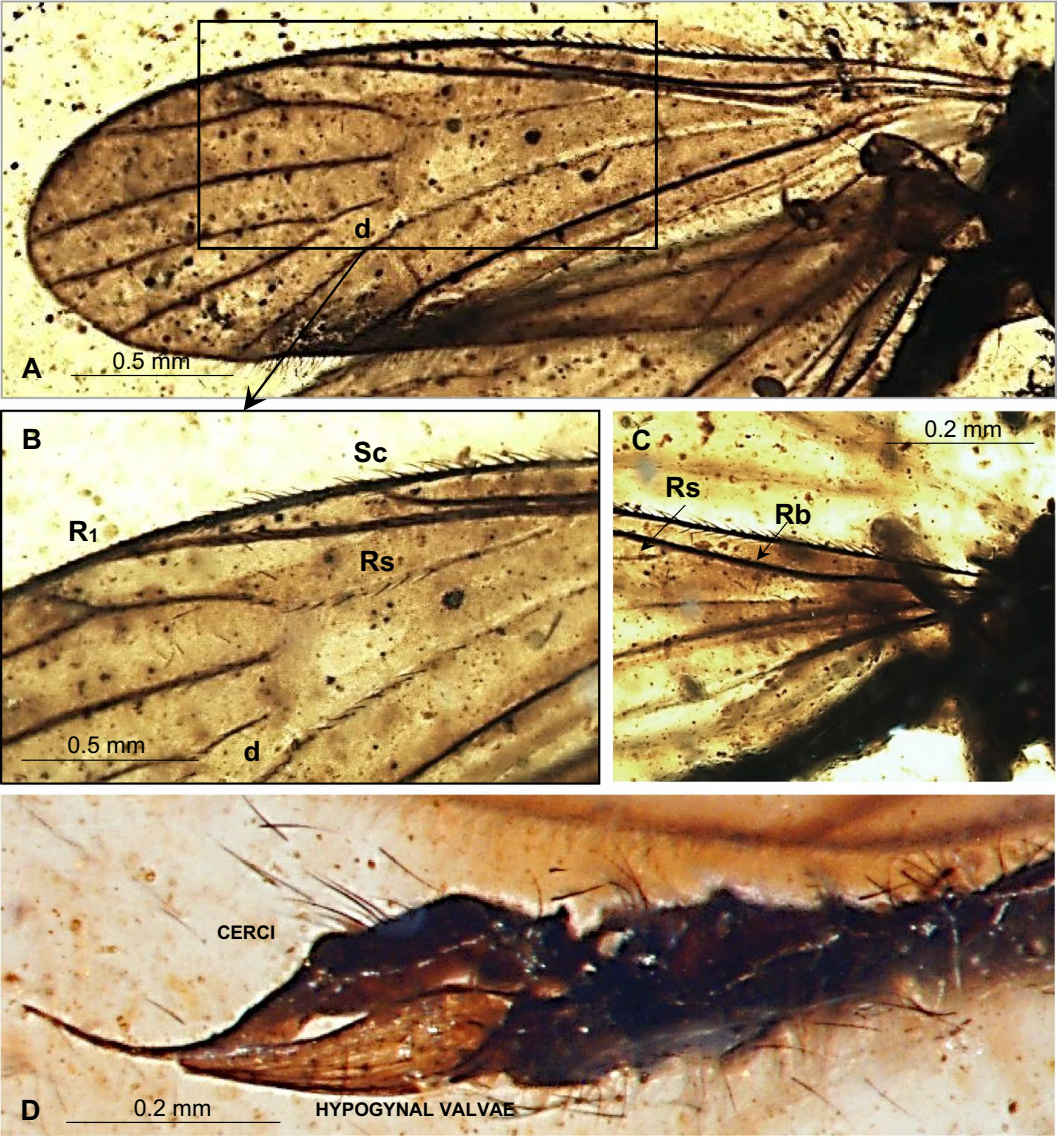
*Gonomyia* (*Iberiana*) *penalveri* subgen. et sp. nov. (Limoniidae), inclusions in Spanish amber: (**A**) wing; (**B**) enlarged view of part of wing; (**C**) base of wing; (**D**) ovipositor.

**Figure 5:**
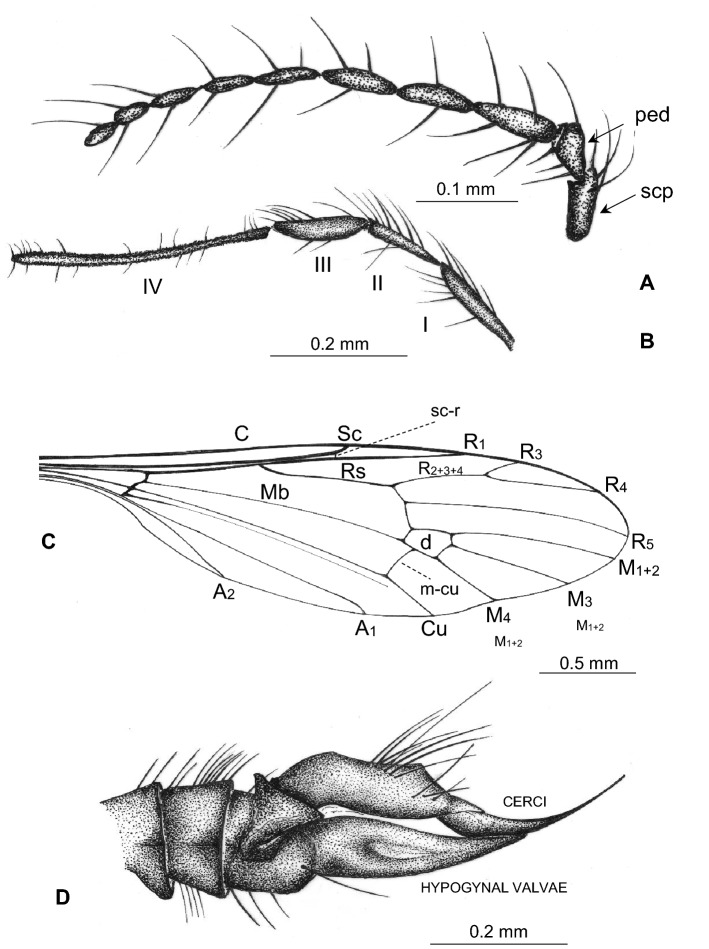
*Gonomyia* (*Iberiana*) *penalveri* subgen. et sp. nov. (Limoniidae), inclusions in Spanish amber: (**A**) antenna; (**B**) palpus; (**C**) wing; (**D**) ovipositor.

*Diagnosis*. As for the subgenus.

*Etymology*. The specific name is dedicated to eminent geologist and paleontologist Enrique Peñalver from the Museo Geominero, Madrid, Spain.

*Material examined*. Holotype No. MCNA 8818 (female), housed in the Museo de Ciencias Naturales de Álava, (Vitoria-Gasteiz, Álava, Spain).

*Horizon and locality*. Lower Cretaceous (Upper Albian); Peñacerrada I amber site (Peñacerrada I, Moraza), eastern area of the Basque-Cantabrian Basin, Burgos, northern Spain.

*Description*. Body (Fig. 2) 2.63 mm long, darkbrown, wings without color pattern, pterostigma absent.

Head (Figs. 2, 3A–C): antenna (Figs. 2, 3A,B, 5A) 0.64 mm long (1/0.08; 2/0.06; 3/0.10; 4/0.09; 5/0.05; 6/0.07; 7/0.05; 8/0.04; 9/0.04; 10/0.06); longer than head, shorter than palpus; scape and pedicel rather short, shorter than first flagellomere; scape cylindrical, narrow, pedicel widened distally, massive, only slightly longer than wider, flagellomeres elongate, oval, flagellomeres 1–5 approximately 3 × as long as wide, flagellomeres 6–8 at most 2 × as long as wide, flagellomeres became more slender to the apex of antenna; last flagellomere shortenest, without elongate setae at apex, first flagellomere with a few elongate setae, approximately as long as segment bearing them, each other flagellomeres with two elongate setae usually longer than segments bearing them; palpus (Figs. 2, 3A,C, 5B) elongate, 0.94 mm long (1/0.19; 2/0.12; 3/0.17; 4/0.46), palpomeres 1–3 elongate, narrow, first palpomere 5 × as long as wide, only slighlty longer than second and third, third palpomere slightly windened in the middle second and third palpomeres 3.5 × as long as wide, last palpomere very elongate and tiny, 2.5 × as long as third one; palpomeres 1–3 with a few elongate setae, only slightly shorter than segments bearing them, last palpomere with several not very elongate setae, approximately equal in length to width of this palpomere.

Thorax (Figs. 2, 3A): scutellum widened in the middle; wing (Figs. 2, 4A–C, 5C) 2.75 mm long, 0.86 mm wide; tip of R_1_ just before fork of R_2+3+4_; sc-r at approximately three of its length from the tip of Sc; Rs 0.65 mm long, longer than half the length of R_5_; R_1_ elongate, ending just beyond d-cell, in approximately 0.25 × the length of R_2+3+4_; R_4_ longer than R_2+3+4_, achieves about 0.6 × the length of Rs; d-cell 0.20 mm long, 1.5 × as long as wide, 0.25 × the length of M_3_; tip of M_4_ just beyond fork of R_2+3+4_ on R_3_ and R_4_; crossvein m-cu almost as long as d-cell; tip of A_2_ just before m-cu; tip of A_1_ just before fork of Rb; haltere (Figs. 2, 3A): stem narrow, elongate, slightly longer than knob.

Abdomen (Figs. 2, 4D): female terminalia – ovipositor (Figs. 4D, 5D) 0.72 mm long, rather short and wide, massive in comparison to the rest of the body, tenth tergite elongate; cercus elongate, tiny, pointed, hypogynal valvae massive, but acutely pointed, tip reaches to middle of cercus.

**Subgenus: *Cretagonomyia*** subgen. nov.

Type species: *Gonomyia* (*Cretagonomyia*) *burmitica* subgen. et sp. nov.

*Diagnosis*. Antenna 13-segmented, longer than palpus; scape and pedicel elongate, comparative length, approximately 2.5 × as long as wide, scape cylindrical, pedicel widened in the middle, first flagellomere elongate, narrow, approximately 4 × as long as wide; palpus shorter than antenna, last palpomere shorter than two penultimate; Sc with its tip just before fork of M_3+4_ on M_3_ and M_4_; elongate and almost straight Rs; R_3_ not very short, approximately 1.5 × the length of R_2+3+4_ and 0.75 × the length of R_4_; basal section of R_5_ separate Rs at an acute angle; vein M_3_ 1.5 × as long as R_2+3+4_; morphology of gonostyles very simple, gonostyles not strongly sclerotized, outer gonostylus narrow, elongate, pointed, longer than inner gonostylus, inner gonostylus as narrow as outer, pointed; A_2_ short, arched.

*Etymology.* The specific name is derived from “creta” (Latin) = Cretaceous and “*Gonomyia*” from nominative genus. Gender: feminine.

*Description*. By monotypy, the description of the subgenus is the same as for the species.

### *Gonomyia* (*Cretagonomyia*) *burmitica* subgen. et sp. nov

(Figs. 6, 7).

**Figure 6:**
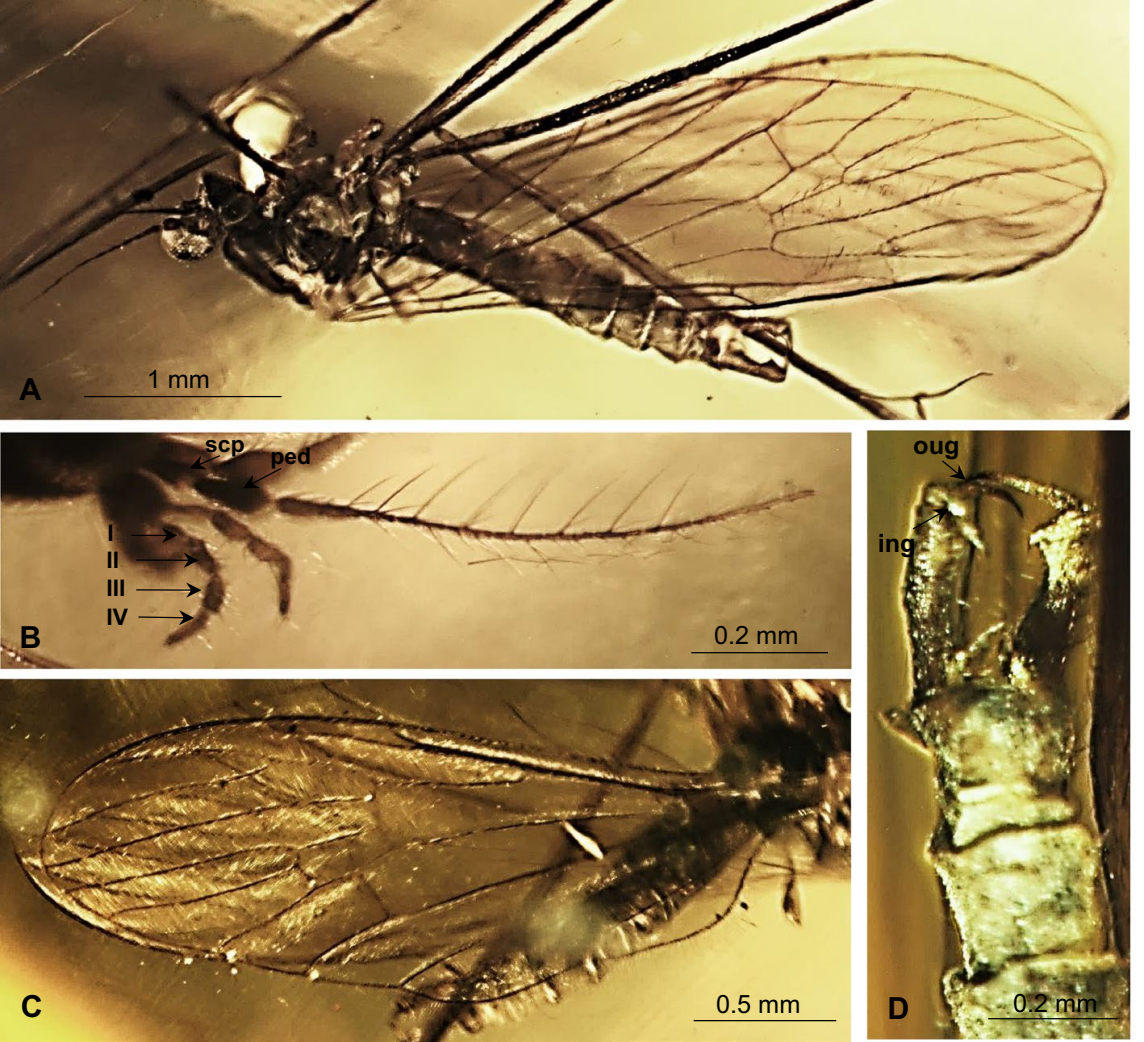
*Gonomyia* (*Cretagonomyia*) *burmitica* subgen. et sp. nov. (Limoniidae), inclusions in Kachin amber: (**A**) body, latero-ventral view; (**B**) antenna and palpi; (**C**) wing; (**D**) hypopygium.

**Figure 7:**
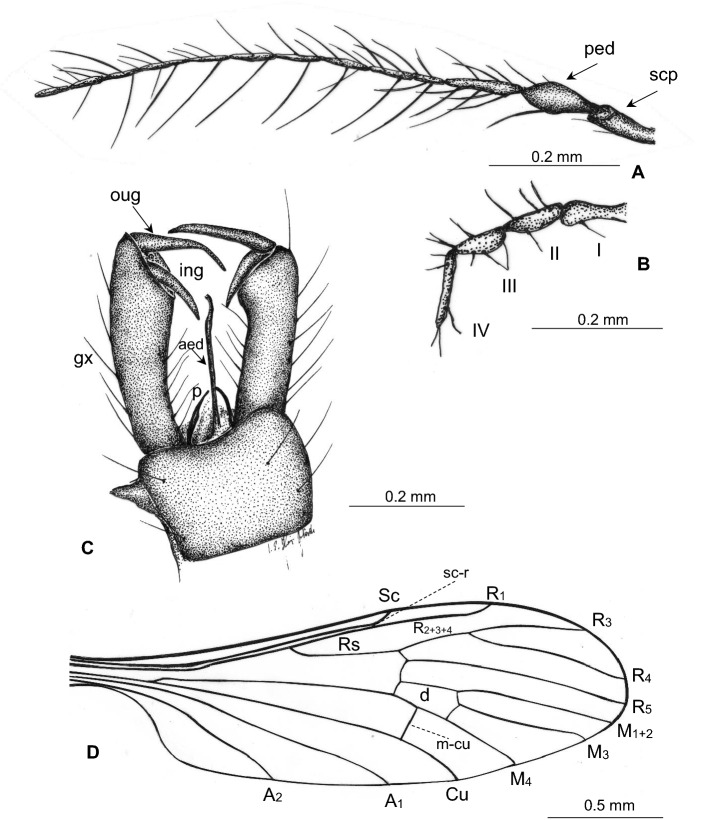
*Gonomyia* (*Cretagonomyia*) *burmitica* subgen. et sp. nov. (Limoniidae), inclusions in Kachin amber: (**A**) antenna; (**B**) palpus; (**C**) hypopygium; (**D**) wing.

*Diagnosis*. As for subgenus.

*Etymology.* The specific name is derived from mineralogical name of the resin containing inclusions—burmite.

*Material examined*. Holotype No. BA02-050 (male), housed in the Nanjing Institute of Geology and Palaeontology, Chinese Academy of Sciences, Nanjing, China.

*Horizon and locality*. Lowermost Cenomanian, Hukawng Valley, northern Myanmar. The mining is done at a hill named Noije Bum, near Tanai Village (26°21′33.41ʺN, 96°43′11.88ʺE).

*Description*. Body (Fig. 6A) 3.02 mm long, brown, wings without color pattern, pterostigma absent.

Head (Fig. 6A): antenna (Figs. 6A,B, 7A) 1.08 mm long (1/0.12; 2/0.10; 3/0.13; 4/0.05; 5/0.05; 6/0.05; 7/0.05; 8/0.05; 9/0.05; 10/0.05; 11/0.05; 12/0.05; 13/0.05); longer than head, longer than palpus; scape cylindrical, narrow, pedicel widened in the middle, massive, approximately 2.5 × longer than wider, flagellomeres elongate, cylindrical, approximately 4 × as long as wide, became more slender to the apex of antenna; last flagellomere as long as penultimate one, without elongate setae at apex, first flagellomere with a few elongate setae, approximately as long as or shorter than segment bearing them, each other flagellomeres excluding the last one with two elongate setae, one of these setae approximately twice as long as segments bearing them, the second one as long or shorter than segments bearing them; palpus (Figs. 6B, 7B) rather short, 0.32 mm long (1/0.10; 2/0.06; 3/0.06; 4/0.10), palpomeres narrow, first palpomere approximately 2.5 × as long as wide, only slightly longer than second and third, third palpomere slightly windened in the middle, second and third palpomeres 2 × as long as wide, last palpomere rather short, only slightly longer than penultimate one, 1.4 × as long as third one; palpomeres with a few not very elongate setae, shorter than segments bearing them.

Thorax (Fig. 6A): scutellum widened in the middle; wing (Figs. 6A,C, 7C) 3.72 mm long, 1.12 mm wide; sc-r at approximately three of its length from the tip of Sc; Rs not very elongate, 0.53 mm long, shorter than half the length of R_5_, R_1_ elongate, ending far beyond d-cell, in approximately 0.2 × the length of R_3_; R_3_ elongate, longer than half the length of R_4_; R_4_ approximately twice as long as R_2+3+4_, longer than Rs; vein M_3_ approximately 2.5 × the length of d-cell; d-cell 0.43 mm long, 2 × as long as wide; tip of M_4_ just before fork of R_2+3+4_ on R_3_ and R_4_; crossvein m-cu shorter than d-cell; tip of A_2_ just before m-cu and fork of Mb; tip of A_1_ far before fork of Rb.

Haltere (Fig. 6A,C): stem narrow, elongate, slightly longer than knob.

Abdomen (Fig. 6A,D, 7D): male terminalia—hypopygium 0.44 mm long, rather short and narrow; gonocoxite narrow, approximately 2.5 × as long as wide; gonostyles narrow, slightly sclerotized, outer gonostylus slightly longer than inner gonostylus, arrange more than 0.3 × the length of gonocoxite, pointed; inner gonostyle widened at base, pointed; aedeagus the average length, parameres short, strongly sclerotized; gonocoxite with not numerous setae, rather short setae.

## Discussion

**Figure 8:**
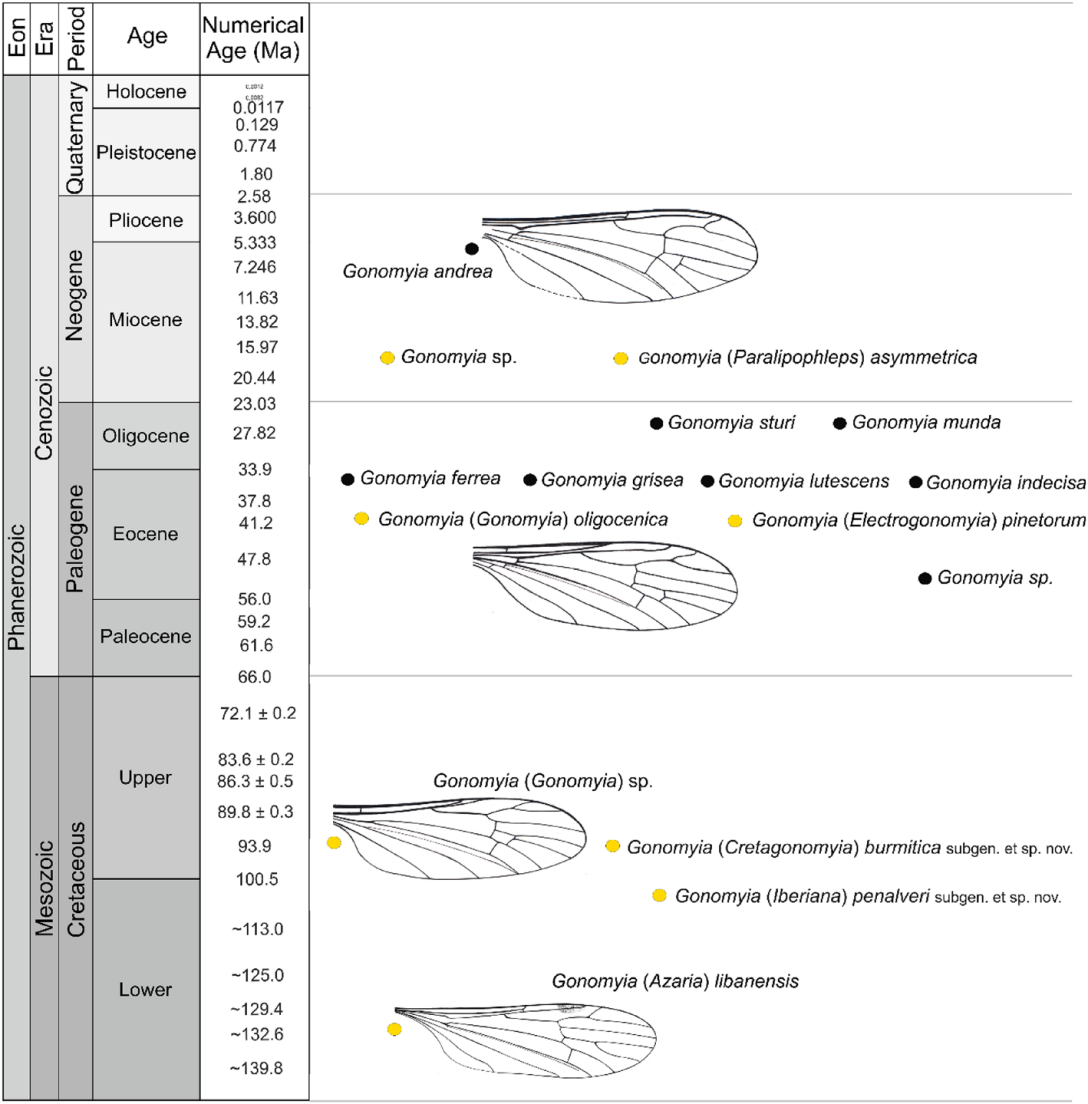
Chronostratigraphic distribution of the *Gonomyia* Meigen^1^ (Diptera: Limoniidae), fossil species.

Though the craneflies of the genus *Gonomyia* are known from many places of different age, and they were preserved as an inclusions in Cretaceous Lebanese or Kachin amber, Eocene Baltic amber and Miocene Dominican amber^9,10,12–14^, or as an imprints are known from area of today’s Europe, from Miocene locality of Italy, Oligocene of Germany or Eocene/Oligocene of England^15–20^ the knowledge about fossil representatives of *Gonomyia* is still insufficient. In recent fauna the genus is diversified and differentiated, while from fossil record only 14 species were known so far, most of them unplaced to any subgenus, two described only as *Gonomyia* sp. (Table 1). From the Creatceous period, from ante-Barremian Lebanese amber is known the oldest representative of *Gonomyia*^10^, and one from Lowermost Cenomanian Kachin amber classified by Podenas and Poinar^12^ to subgenus *Gonomyia*. The discovery of new materials give us new information very important for understanding the earliest stage of evolution of the genus *Gonomyia*. Finding representatives of the genus first in Lebanese amber, and then in Spanish or Kachin amber may indicate that the genus was already widespread on Earth in the Cretaceous. Moreover, comparison of known and new materials show that the genus was probably diversified in this period. Known from the Lebanese amber species belongs to subgenus *Azaria*, a lineage that is not represented either in the modern fauna or in the fossil record of other periods in the history of the Earth (Fig. 8). Characteristic position of crossvein m-cu behind the bifurcation of Mb into M_1+2_ and M_3+4_ occur in *Azaria*. Regardless of whether or not it is plesiomorphic character, this characteristic still persists in the present in taxa of several subgenera (e.g. *Gonomyia* and *Teuchogonomyia*). Such features as long vein R_2+3+4_, crossvein r–r (R_2_) atrophied, relatively short vein R_3_ or narrow d-cell allowed to classify the newly described herein species to the genus *Gonomyia*. Moreover, the study new peculiar materials allowed to establish two new subgenera with differentiated morphology of wing venation and number of flagellomeres. The subgenus *Iberiana* subgen. nov. is characterized for example by ten number of antenna, or small and almost rhomboidal d-cell, while the gonostyles in subgenus *Cretagonomyia* are very simple, only slightly sclerotized without any processes. But, both of them characterize by the occurence of crossvein m-cu in the position shifted beyond fork of Mb, but in contrast to the subgenus *Azaria* d-cell in these two subgenera is closed. This location of m-cu indicate the ancestral position of these subgenera within the genus *Gonomyia*, same as for the subgenus *Azaria*^10^.

One more species have been described from the Cretaceous period^12^. Based on inclusion of female body in Cretaceous Kachin amber Podenas and Poinar^12^ described one new species which classified to the subgenus *Gonomyia* and there is no doubt that these two different species represents two different subgenera. The differences between *G.* (*I.*) *penalveri* subgen. et sp. nov. are well visible not only in morphology of antenna or wing venation, but also in the structures of ovipositor. Ovipositor of species described by Podenas and Poinar^12^ is uniformly light brown with tenth tergite elongate, comparatively short cercus with upturned tip and hypogynial valve are long, acutely pointed, tip of this structure reaches to middle of cercus. In *G.* (*I.*) *penalveri* sp. nov. dark brown ovipositor is equipped with elongate tenth tergite, cercus is long and tin pointed and hypogynial valvae are long, acutely pointed, of its tip also reaches to middle of cercus, but ovipositor is rather short and massive in comparison to the rest of the body. Some significant differences are also visible in morphology of antenna, palpus and wing venation. In *G.* (*I.*) *penalveri* sp. nov. antenna is 10-segmented with two elongate setae on each flagellomere, usually longer than segment bearing them, while in *G.* (*G.*) sp. antenna is 16-segmented (what is characteristic for subgenus *Gonomyia*) with setae shorter than length of flagellomeres. In *G.* (*I.*) *penalveri* sp. nov. palpomeres are elongate, approximately 3 × as long as wide, while in *G.* (*G.*) sp. at most 2 × as long as wide. In *G.* (*I.*) *penalveri* sp. nov. three basal palpomeres are elongate, 3 × to 5 × (first palpomere) as long as wide, last palpomere is equal in length preceding palpomeres taken together, in *G.* (*G.*) sp. three basal palpomeres are short, approximately equal in length, their length only slightly exceeds their width, terminal palpomere is elongate, cylindrical, about as long as two preceding palpomeres taken together. In newly described species d-cell is rather romboidal, in *G.* (*G.*) sp. is almost rectangular, vein R_2+3+4_ in *G.* (*I.*) *penalveri* sp. nov. is elongate, arrange 2.5 × the length of R_3_ but is shorter than R_4_, R_3_ is short, and reach only 0.3 × the length of R_4_, in *G.* (*G.*) sp. vein R_2+3+4_ is rather short, corresponds to 1.5 × the length of R_3_, and half the length of R_4_. Tip of R_1_ is situated just before fork of R_2+3+4_ in *G.* (*I.*) *penalveri* sp. nov., in *G.* (*G.*) sp. just beyond fork of Rs. Basal section of R_5_ separate Rs almost at right angles in *G.* (*I.*) *penalveri* sp. nov., while in *G.* (*G.*) sp. is elongate and connected to Rs at an acute angle.

In recent fauna the genus *Gonomyia* is reach in species and widespread from cold to tropical zones (except for Antarctica). Although the genus is the most diversified in species in the Neotropic (202 species and subspecies), in this region is represented by four subgenera—*Gonomyia*, *Leiponeura*, *Neolipophleps* and *Paralipophleps*, while in Nearctic occur only 40 species and subspecies within six subgenera—*Gonomyia*, *Idiocerodes*, *Leiponeura*, *Neolipophleps*, *Paralipophleps* or *Teuchogonomyia*^2^.

In the ancient ages of the Earth’s history, the genus *Gonomyia* was probably also rich in species and widespread, as evidence by the presence of representatives in many different age fossil resins and in sediments from various places around the world, even if they are single species. The Cretaceous period in the history of the Earth is of great importance due to changes in fauna and flora, but also climate change. The resin from which the Spanish amber was produced in subtropical forests with seasonal wet-dry climate dominated by conifers and inhabited by ferns, gymnosperms and early angiosperms^26^. Kachin amber deposits as considered Grimaldi et al.^27^ were formed in tropical environment within an average temperature range of 32–55 °C, Lebanese amber formed in a tropical or subtropical, moderate to hot and very wet, dense forests^28^. In recent fauna representatives of *Gonomyia* are rather connected with flowing and standing waters. For example larvae of *Gonomyia* (*Gonomyia*) *abscondita* Lackschewitz^29^ are often found near the shores of flowing and standing waters, the species occur near streams and water margins on neutral acidic soil, frequently in wet woodland or scrub. Imago of this species usually occur in springs and headwater streams^26^. The larvae of the other species of the same subgenus as *Gonomyia* (*Gonomyia*) *conoviensis* Barnes^30^ are probably semi-aquatic, associated with sheltered wooded streams in upland areas, as well as other habitats in coastal locations, often occured with small streams in upland woodland situations. Larvae of *Gonomyia* (*Gonomyia*) *hippocampi* Stubbs and Geiger^31^ can be found in marginal situations along flowing and standing waters. Also other subgenera within *Gonomyia* are associated with the aquatic, wetland environment as the representatives of subgenus *Leiponeura* were found in the middle reaches of the river, their larvae occur also in saturated earth that ranges from coarse and sand to fine silit, but are much more frequent and aboundant in distinctly sandy situations. And although representatives of the subgenus *Prolipophleps* can be a specialist indicator of calcareous soil or bedrock, they are found in riverine sediments, mixed broadleaved forests along stream^2^.

The discovery of representatives of *Gonomyia* in these three Cretaceous resins testifies that this genus must have been diversified and widespread in the past. Moreover, these insects were adapted to tropical and subtropical palaeohabitats already in the Cretaceous period, as evidenced by the origination environment of Lebanese amber, Kachin and Spanish amber.

## Material and methods

The study material—specimen No. MCNA 8818 comes from Cretaceous amber deposit of Peñacerrada I (Álava amber), is dated to Upper Albian (105 Ma^21^), are located in the northern slope of Sierra de Cantabria, in the southern limit of the Basque-Cantabrian Basin (northern Spain)^26^. Amber occurs in lutitic layers of deltaic origin with abundant coal. The specimen is housed in the Museo de Ciencias Naturales de Álava, (Vitoria-Gasteiz, Álava, Spain) (Fig. 1A–D). The specimens were embedded in epoxy resin (EPO-TEK 301) as described Corral et al.^32^ and Nascimbene and Silverstein^33^, which allowed physical protection and optimal study in ventral, lateral and dorsal views. The piece of amber has meassures of 8 × 6 × 1.5 mm and is embebbed in a resin piece of 10 × 8 × 1.5 mm.

The specimen No. BA02-050 (male) was found as an inclusion in the Cretaceous Kachin amber which deposits comes from the Hukawng Valley in the northern Myanmar, Myitkyina and Upper Chindwin districts (Myanmar)^34–37^ and are dated on 98.79 ± 0.62 Ma according to Shi et al.^38^ (data received based on research of zircons from the amberbearing bed), therefore, the amber is likely to be of early Cenomanian age^39^.

The specimen is housed in the Nanjing Institute of Geology and Palaeontology, Chinese Academy of Sciences, Nanjing, China.

The specimens were examined with a Nikon (SMZ25) stereomicroscope Nikon SMZ 1500 equipped with a Nikon DS–Fi1 camera. The measurements were taken with NIS–Elements D 3.0 software. The length of the discal cell—measurements were given from its posterior edge to the point of connection of vein m-m with vein M_3_. The measurements were given only for undamaged structures. Drawings were completed by tracing the specimen and photographs, were made by Iwona Kania-Kłosok. Map (Fig. 1C) was built using the map Maps-For-Free (https://maps-for-free.com), map (Fig. 1D) was built using the plate tectonic reconstruction (https://www.odsn.de), both were modified with the software programs Corel Draw and Corel Photopaint X7. Abbreviations in accordance with: A_1_—first anal vein; A_2_—second anal vein; ant—antennae; Cu—cubital vein; d—discal cell; gx—gonocoxite; ing—inner gonostylus; M_1_–M_4_—first to fourth medial vein; m-cu—medio-cubital crossvein; Mb—medial-basal vein; oug—outer gonostylus; ovip—ovipositor; p—paramere; pl—palpi; ped—pedicel; r–r (R_2_)—second radial vein; R_3_–R_5_—third to fifth radial veins; Rb—radial-basal vein; Rs—radial sector vein; Sc—subcostal vein; sc-r—subcostal crossvein; scp—scape; I–IV—palpomeres first to fourth; Fig. 8: red circle—deposits of amber inclusion; yellow circle—deposits of imprint. The wing venation nomenclature follows that of McAlpine^40^.

## Data Availability

All data generated or analyzed during this study are included in this published article.
